# Influence of Anion Structure on Thermal, Mechanical and CO_2_ Solubility Properties of UV-Cross-Linked Poly(ethylene glycol) Diacrylate Iongels

**DOI:** 10.3390/membranes10030046

**Published:** 2020-03-17

**Authors:** Ana P. S. Martins, Asier Fdz De Añastro, Jorge L. Olmedo-Martínez, Ana R. Nabais, Luísa A. Neves, David Mecerreyes, Liliana C. Tomé

**Affiliations:** 1 POLYMAT, University of the Basque Country UPV/EHU, Joxe Mari Korta Center, Avda. Tolosa 72, 20018 Donostia-San Sebastian, Spain; 2 Instituto de Tecnologia Química e Biológica António Xavier, Universidade Nova de Lisboa, 2780-157 Oeiras, Portugal; 3 LAQV-REQUIMTE, Chemistry Department, Faculdade de Ciência e Tecnologia, Universidade Nova de Lisboa, 2829-516 Caparica, Portugal; 4 Ikerbasque, Basque Foundation for Science, E-48013 Bilbao, Spain

**Keywords:** ionic liquids, fluorinated and cyano-based anions, UV cross-linked polymer network, Iongels, CO_2_ solubility, CO_2_/N_2_ separation performance

## Abstract

Iongel-based CO_2_ separation membranes were prepared by fast (< 1 min) UV-initiated polymerization of poly(ethylene glycol) diacrylate (PEGDA) in the presence of different ionic liquids (ILs) with the [C_2_mim]^+^ cation and anions such as [TFSI]^−^, [FSI]^−^, [C(CN)_3_]^−^ and [B(CN)_4_]^−^. The four ILs were completely miscible with the non-ionic PEGDA network. Transparent and free-standing iongels containing between 60 and 90 %wt of IL were obtained and characterized by diverse techniques (FTIR, TGA, DSC, DMTA, SEM, CO_2_ solubility and pure gas permeability). The thermal and mechanical stability of the iongels, as well as CO_2_ solubility, were found to be strictly dependent on the IL content and the anion’s nature. The TGA results indicated that the iongels mostly follow the thermal profile of the respective neat ILs. The DMTA analysis revealed that the iongels based on fluorinated anions have higher storage modulus than those of cyano-functionalized anions. Conversely, the PEGDA–C(CN)_3_ iongels presented the highest CO_2_ solubility values ranging from 72 to 80 mmol/g. Single CO_2_ permeabilities of 583 ± 29 Barrer and ideal CO_2_/N_2_ selectivities of 66 ± 3 were obtained with the PEGDA–70 C(CN)_3_ iongel membrane. This work demonstrates that the combination of PEGDA with high contents of the best performing ILs is a promising and simple strategy, opening up new possibilities in the design of high-performance iongel membranes for CO_2_ separation.

## 1. Introduction

The potential of using ionic liquid (IL) materials to improve membrane-based CO_2_ separation processes has been demonstrated in recent years [1,2,3]. In particular, significant research efforts have been made towards the development of iongels, also called ion gel or ionic liquid gel membranes, which are soft solid materials comprised by a dispersed ionic liquid phase and a solid continuous phase [4]. The intrinsic IL properties are preserved in the iongels, making them a suitable strategy to prepare pseudo-solid membranes with CO_2_ separation performances close to those of supported ionic liquid membranes (SILMs), while unveiling improved mechanical strength and higher burst pressures [1]. 

Several strategies have been used in the preparation of iongel-based CO_2_ separation membranes, such as the use of low-molecular-weight gelators [5,6] or triblock copolymers [7,8], the blending of ILs with non-charged polymers [9,10] or poly(ionic liquid)s (PILs) [11,12,13,14], and UV-initiated free radical polymerization of acryl or vinyl IL monomers in the presence of free (non-polymerizable) ILs [15,16,17,18]. Particular attention has also been paid to cross-linked iongels based on polymer networks, since this strategy allows for the fabrication of membranes with high free IL contents, while maintaining solid-like mechanical properties [19]. For instance, Carlisle et al. [20] developed a “curable PIL” platform and prepared cross-linked PIL/IL iongels that contained ≥ 80 wt % of free [C_2_mim][TFSI] IL and had single-CO_2_ permeabilities of 500 ± 60 Barrer and ideal CO_2_/N_2_ selectivities of 24 ± 4. The inclusion of polymerizable pendant groups onto the PIL backbone revealed distinct advantages for producing thin-film composite membranes, as the curable PILs exhibited faster gelation rates and less solution penetration into porous supports compared to cross-linkable IL monomer systems [20]. MacDanel et al. [21,22] proposed another approach making use of step-growth polymerization to produce epoxy-amine-based cross-linked PIL networks and PIL/IL iongel membranes. The results showed that increasing the free [C_2_mim][TFSI] IL content (50, 60, 75 wt %) increases the single CO_2_ permeabilities from 100 ± 5 to 510 ± 20 Barrer, while slightly increasing the ideal CO_2_/N_2_ selectivity from 28 to 35. In addition, Matsuyama and coworkers focused on the concept of double-network (DN) iongel membranes containing two asymmetric polymer networks, one as a rigid and tightly cross-linked polyelectrolyte and another as a soft and ductile cross-linked polymer [23,24,25]. Some of the prepared DN iongels, with ca. 80 wt % of phosphonium-based ILs combining either the [Pro]^−^ or [Inda]^−^ anions, not only sustained large compressive stress (more than 25 MPa), but also showed CO_2_ and N_2_ permeabilities comparable to those of the corresponding SILMs. Despite all the progress and the good results obtained, the preparation routes of these iongels involve either multi-pot polymerization procedures or a number of organic syntheses/purification steps at the PIL or IL monomer level. 

One promising way to overcome the multi-step synthesis is to create iongels in a single-pot by in situ thermal or UV-initiated polymerization of commercially available non-ionic polymer networks in the presence of a free IL [26,27,28]. Bearing in mind that cross-linked poly(ethylene glycol) (PEG) iongels have been widely explored as solid electrolyte materials [29,30,31], and considering the good CO_2_/N_2_ selectivity of PEG-based membranes [32,33], further attention has recently been given to PEG-based cross-linked iongel membranes for CO_2_ separation. Kusuma et al. [34] studied the phase miscibility of a variety of ILs at moderate loadings (40 vol.%) into two different cross-linked polymer matrices, poly(ethylene glycol) diacrylate M_n_ 700 g mol^−1^ (PEGDA) and a PEGDA copolymer with a thiol-functionalized polysiloxane. The ILs consisting of an aromatic cation with acid proton (imidazolium and pyridinium) and a low basicity anion ([TFSI]^−^) imparted the most stable and miscible iongel membranes and led to significant increases in gas permeability [34]. Later on, Kusuma et al. [35] extended their study by incorporating a series of 1,3-substituted imidazolium [TFSI] ILs (at 40 and 60 vol.% loading) into a polymer network consisting of two monomers, the ethoxylated (20) trimethylolpropane triacrylate and the 3,6-dioxa-1,8-octanethiol. The results demonstrated that terminal substituents such as hydroxyl, benzyl, nitrile or trimethylsilyl can decrease chain mobility and/or reduce the free volume cavity site, while short alkyl or alkoxy chains can significantly increase gas permeability through the iongels studied, with [C_2_mim]^+^ being the most effective cation [35]. Furthermore, Deng et al. [36] incorporated ILs containing the [C_4_mim]^+^ cation and different anions ([TFSI], [BF_4_], [PF_6_] and [C(CN)]_3_) into PEGDA membranes with interpenetrating networks based on aza-Michael addition and acrylate homopolymerization. The presence of ILs afforded superior gas transport properties at high IL content (80 wt %) and the [C_4_mim][C(CN)]_3_ disclosed the best CO_2_ separation performance, with a CO_2_ permeability of 134 Barrer and a CO_2_/N_2_ selectivity of 49.5 [36]. 

**Figure 1:**
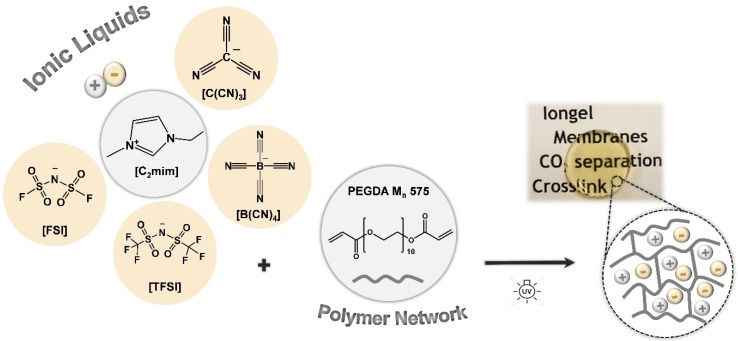
Chemical structures of the ionic liquids (ILs) and polymer network (PEGDA) used in this work.

In this work, iongels based on cross-linked PEGDA and four different ILs, combining the [C_2_mim]^+^ cation and anions such as [TFSI]^−^, [FSI]^−^, [C(CN)_3_]^−^ and [B(CN)_4_]^−^ (Figure 1), were prepared with IL contents between 60 and 90 wt %. The influence of both the IL content and IL anion on the iongels’ chemical structure, thermal and mechanical stability, as well as CO_2_ solubility, was systematically investigated by various characterization techniques. Single-gas permeation measurements were also conducted to evaluate the potential of the prepared iongels as CO_2_ separation membranes. The choice of the IL anions was dictated by the high CO_2_ permeabilities and selectivities previously obtained through SILM configurations [37,38,39]. Although cross-linked PEGDA membranes demonstrated improved gas transport properties when longer PEGDA (M_n_ 700 g mol^−1^) monomer was used [36], a shorter PEGDA monomer (M_n_ 575 g mol^−1^) was selected for this study, mainly due to the higher cross-linking density of the resulting polymer network, so that iongels with high IL contents and self-standing properties could be obtained. 

## 2. Experimental Section

### 2.1. Materials

The poly(ethylene glycol) diacrylate (PEGDA, M_n_ 575 g mol^−1^) and 2-Hydroxy-2-methylpropiophenone (DAROCUR, 97 wt % pure) were purchased from Sigma-Aldrich (Spain). The ionic liquids 1-Ethyl-3-methylimidazolium bis(fluorosulfonyl)imide ([C_2_mim][FSI], 99.5 wt % pure) and 1-ethyl-3-methylimidazolium tetracyanoborate ([C_2_mim][B(CN)_4_], 98 wt % pure) were supplied by Solvionic (Toulouse, France) and Merck KGaA (Madrid, Spain), respectively. IoLiTec GmbH (Heilbronn, Germany) provided the 1-ethyl-3-methylimidazolium bis(trifluoromethylsulfonyl)imide ([C_2_mim][TFSI], 99 wt % pure) and 1-ethyl-3-methylimidazolium tricyanomethanide ([C_2_mim][C(CN)_3_], 98 wt % pure). Chemical structures of the ionic liquids (ILs) and the polymer network (PEGDA) are illustrated in Figure 1. Carbon dioxide (CO_2_, 99.998 wt % pure) and nitrogen (N_2_, 99.99 wt % pure) gases were supplied by Praxair (Almada, Portugal).

### 2.2. Preparation of UV-Cross-Linked PEGDA Iongels

**Figure 2:**
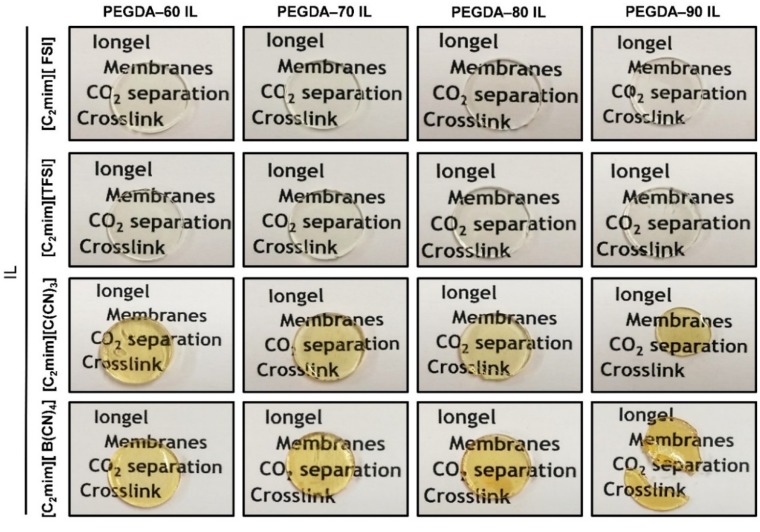
Images of the prepared UV-cross-linked poly(ethylene glycol) diacrylate (PEGDA) iongels.

The cross-linked PEGDA iongels containing different amounts of the selected ILs (60, 70, 80 and 90 wt %) were prepared by UV-initiated free radical polymerization. First, calculated amounts of IL, poly(ethylene glycol) diacrylate (PEGDA), and 2-hydroxy-2-methylpropiophene (DAROCUR) as a radical photoinitiator (5 wt % to PEGDA) were magnetically mixed in a glass vial, at room temperature, until homogenous solutions were obtained. All the prepared solutions were degassed for few minutes in order to eliminate possible bubbles. The mixtures were then casted onto silicone molds and exposed to UV light using a Dymax UVC-5 UV Curing Conveyor System with 800 mW cm^−2^, operating at wavelength of 365 nm. The distance from sample to lamp was 10 mm and the belt speed was fixed at 1 m∙min^−1^. Three repetitive cycles were applied to accomplish full photopolymerization. After irradiation, the materials were kept in a vacuum oven overnight at 60 °C. The resulting iongels were carefully peeled out from the molds and characterized. Figure 2 depicts images of the prepared UV-cross-linked PEGDA iongels. Given that all the iongels were prepared with PEGDA and [C_2_mim]-based ILs, the iongel samples are systematically designated as “PEGDA–XX anion” according to the IL content (XX), and then followed by the acronym of IL anion, as depicted in Table 1.

### 2.3. Characterization Methods

Total Reflection Fourier Transform Infrared Spectroscopy (ATR-FTIR) was performed to confirm the success of the photopolymerization, as well the chemical structures of the prepared iongels. All spectra were collected using a Bruker ALPHA spectrometer from 400 to 4000 cm^−1^. The resolution was 4 cm^−1^ after 24 scans. Samples from the pre-crosslinked solutions and iongels were placed directly on the ATR crystal.

Thermogravimetric Analyses (TGA) were carried out on a TGA Q50 device from TA instruments. The samples (ca. 10 mg) were heated at a constant rate of 10 °C min^−1^, from room temperature to 600 °C, under nitrogen atmosphere to prevent thermo-oxidative degradation of the samples. The Universal Analysis software (version 4.5 A) was used to determine the onset (*T_onset_*) and decomposition (*T_dec_*) temperatures.

Differential Scanning Calorimetry (DSC) experiments were performed on a Perkin Elmer 8500 DSC equipped with an Intracooler III. Samples (ca. 5 mg) were crimped in non-recyclable aluminum hermetic pans and analyzed under nitrogen atmosphere by heating and cooling cycles at a rate of 20 °C min^−1^. First, the samples were heated from 25 to 150 °C and kept isothermally for 3 min at 150 °C to erase thermal history. Subsequently, the samples were cooled down to −70 °C and kept isothermal for 10 min. Second-run heating cycles were conducted and used to further investigate the phase transition behavior of all samples. 

The mechanical properties of the iongels were assessed by Dynamic Mechanical Thermal Analysis (DMTA). The measurements were conducted in compressing mode on a Triton 2000 DMA device (Triton Technology). Circular samples (11 mm of diameter) were heated from 10 to 100 °C at a constant rate of 4 °C min^−1^ and a frequency of 1.0 Hz.

Scanning Electron Microscopy (SEM) micrographs of the fractured iongels were obtained on a Hitachi TM3030 tabletop microscope (Hitachi High-Technologies). Samples were coated with a gold (Au) thin layer. 

### 2.4. CO_2_ Solubility Measurements

CO_2_ coefficient solubilities for the iongels, neat ILs and cross-linked PEGDA were determined at 30 °C using the pressure decay method adapted from [40]. Briefly, a sample (ca. 0.5 g) was placed in the absorption compartment and then exposed to a CO_2_ atmosphere. The pressure decay with time, which is related to the absorption of the gas by the sample, was monitored using a pressure transducer (Druck PCDR 910 model 99166, UK) until it reaches the saturation state. The CO_2_ solubility coefficient was then calculated as the number of moles of gas absorbed by the amount of sample (mol_CO2_/g_sample_).

### 2.5. Gas Permeability Experiments

Single-gas CO_2_ and N_2_ permeation measurements were performed using a gas permeation setup, fully described elsewhere [41]. Each experiment started by pressurizing both compartments with a pure gas and, after the pressure was stabilized, a transmembrane driving force of around 0.7 bar of relative pressure between the compartments was established. In order to ensure a constant temperature throughout the experiments, the permeation cell was placed in a thermostatic water bath (Julabo, model EH, Germany), where the temperature was set at 30 °C. The pressure variation in each compartment over time was monitored by two pressure transducers (Druck PCDR 910 models 99166 and 991675, UK). Pressure monitoring and data acquisition were controlled by an in-house developed software. The pure gas permeabilities were determined for PEGDA–70 C(CN)_3_ supported on Sartorius^TM^ polyamide (PA) membrane filter supplied by Fisher Scientific (Spain), with a pore size of 0.2 µm. First of all, the PA membrane filter was placed on top of a quartz plate. The iongel precursor mixture was slowly drop-casted onto the PA filter using a silicon frame as a spacer. Then, a second identical quartz plate was positioned on top of the mixture and compressed using staples to hold the two plates together. Finally, the plates were exposed to UV light and the resulting membrane was carefully removed from the glass plates. The average thickness (300 µm) of the supported PEGDA–70 C(CN)_3_ iongel membrane was calculated before and after testing using a micrometer. 

The permeability of a pure gas through a membrane was calculated from the obtained pressure data measured over time, on both compartments, according to Equation (1)

(1) $$\frac{1}{\beta }\ln\frac{p_{feed_{0}}-p_{perm_{0}}}{p_{feed}-p_{perm}}=\frac{1}{\beta }\ln\frac{∆p_{0}}{∆p}=P\frac{t}{l}$$

where $p_{feed}$ and $p_{perm}$ correspond to the pressure in the feed and permeate compartments (bar), respectively, *P* is the membranes’ permeability (m^2^ s^−1^, where 1 Barrer = 8.3 × 10^−13^ m^2^ s^−1^), *t* is the time (s), *l* is the mean membrane thickness (m) and *β* represents a geometric parameter (m^−1^), given by Equation (2),

(2) $$\beta =A\;\left(\frac{1}{V_{feed}}+\frac{1}{V_{perm}}\right)$$

where *A* is the membrane’ area (m^2^) and $V_{feed}$ and $V_{perm}$ correspond, respectively, to the volume of the feed and permeate compartments (m^3^). Pure gas permeability results were obtained from the slope when plotting 1/*β* ln($∆p_{0}$/∆p) as a function of *t*/*l*.

The ideal selectivity of each membrane was calculated from the ratio of the permeabilities of the two different gases, according to Equation (3),

(3) αCO2N2=PCO2PN2

## 3. Results and Discussion 

Several iongel membranes were prepared by in situ UV-initiated free radical polymerization of PEGDA within different amounts (60, 70, 80, 90 wt %) of each of the four ILs depicted in Figure 1. The ILs investigated in this work were completely miscible with liquid PEGDA at room temperature, as evidenced by the optical transparency of their mixtures. The photopolymerization process was very fast (<1 min) and solid materials were formed, as confirmed by the optical appearance of the iongel membranes shown in Figure 2. Free-standing iongel membranes, homogeneous and flexible to be easily handled, were obtained up to 80 wt % IL content. On the other hand, the iongel membranes with 90 wt % IL were too soft to be handled and easily broke, probably due to their lower cross-link density, which imparts reduced mechanical integrity. Different techniques were employed to characterize the iongels prepared, including FTIR, TGA, DSC, DMTA, SEM and CO_2_ solubility. Except for the gas permeability tests, all the other characterization experiments were performed on neat iongel membranes, without the PA porous support.

### 3.1. FTIR Analysis

**Figure 3:**
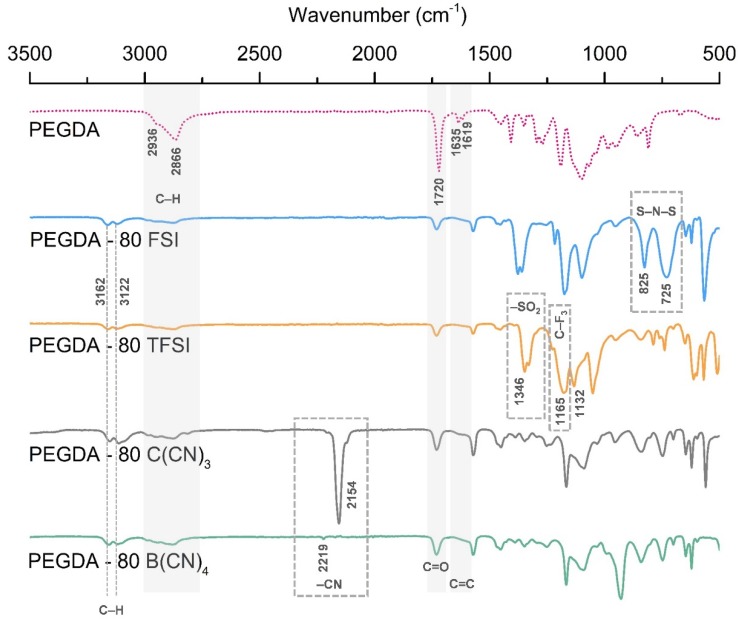
FTIR spectra of the pristine PEGDA and iongels (80 wt % IL) bearing different anions.

FTIR spectroscopy was used to confirm the chemical structure of the prepared iongels, as well as to observe the variation in the peaks associated with the reactive acrylate double-bond on the PEGDA-based polymer network. The obtained FTIR spectra of the pristine PEGDA and the iongels containing 80 wt % of the different ILs studied are depicted in Figure 3. 

The FTIR spectra obtained for PEGDA are similar to those previously published in the literature [36,42]. The presence of PEGDA in the iongels can be confirmed by the peaks observed at 1720 cm^−1^, attributed to the C=O symmetric stretching and the bands at around 2866 and 2936 cm^−1^, assigned to C-H stretching. It can also be seen that the vibration bands at 1635 cm^−1^ (C=C symmetric stretching) and 1619 cm^−1^ (C=C asymmetric stretching), arising from the presence of terminal acrylate groups, are hardly observed or completely disappeared in the formed iongels. This indicates the high extent of the photopolymerization process of acrylate from PEGDA, which is recognized for its ability to build cross-linking networks. 

Considering that the studied ILs have a common imidazolium cation ([C_2_mim]^+^) in their structures (Figure 1), the characteristic absorption bands of the imidazole ring can be observed for all iongels (Figure 3) at around 3122 and 3162 cm^−1^, which are attributed to C-H stretching modes [43,44]. The description of the peaks related to the different IL anion structures is given as follows 725 cm^−1^ (S–N–S symmetric stretching) and 825 cm^−1^ (S–N–S asymmetric stretching) associated with the [FSI]^–^ anion [45]; 1134 cm^−1^ (–SO_2_ asymmetric stretching), 1165 cm^−1^ (C–F_3_ asymmetric stretching) and 1346 cm^−1^ (–SO_2_ asymmetric stretching) attributed to the [TFSI]^–^ anion [43]; 2154 and 2219 cm^−1^ (–CN stretching vibrations) corresponding to the [C(CN)_3_]^–^ and [B(CN)_4_]^−^ anions, respectively [12]. 

Depending on the IL content, differences in the intensity of the absorption bands associated to the imidazole ring and the different anions were also observed. In general, the peaks of the ILs become more evident in the spectra of the iongels when increasing the IL content (see Supplementary Materials for further details, Figures S1–S4), indicating that the ILs incorporated into the polymer network do increase as expected. Nevertheless, no significant deviations in the abovementioned absorption bands were detected in the iongels spectra compared to those of ILs. 

### 3.2. Thermal Analysis

The degradation profiles of the cross-linked PEGDA, neat ILs and respective iongels, were assessed by TGA analysis, aiming not only to determine their decomposition temperature, but also to study the influence of the different IL anions on the thermal stability of the prepared iongels. The onset (*T_onset_*) and decomposition (*T_dec_*) temperatures are given in Table 1, while the TGA thermograms of the iongels containing different amounts (from 60 to 90 wt %) of the studied ILs are provided as Supplementary Material (Figure S5).

The TGA profiles obtained for the cross-linked PEGDA and neat ILs are in agreement with what has been reported in the literature [12,46,47,48,49]. The iongels mostly follow the thermal profile of the respective neat ILs (Figure S5), since the prepared materials have a larger IL content (> 60 wt %) in their composition. Among the ILs studied, the [C_2_mim][TFSI] presents the highest *T_onset_* (424 °C) and *T_dec_* (463 °C), while the [C_2_mim][FSI] has the lowest *T_onset_* (294 °C) and *T_dec_* (343 °C). The same behavior is observed for the prepared iongels (Table 1), wherein PEGDA–TFSI materials exhibit the highest thermal stabilities and the PEGDA–FSI are the less thermally stable iongels. Nevertheless, all the prepared iongel samples fit in terms of thermal stability (*T_onset_* values ranging from 231 to 364 °C) to be used for post-combustion CO_2_ separation processes, which are typically performed at temperatures of around 100 °C. 

**Figure 4:**
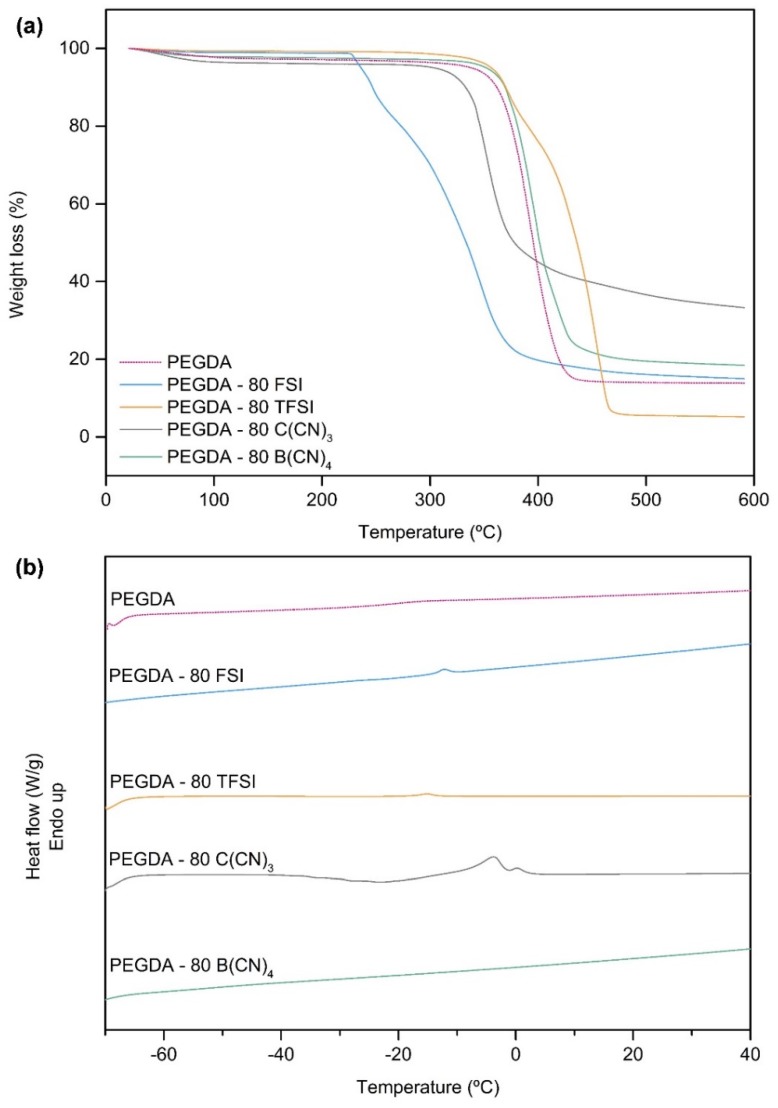
(**a**) Thermogravimetric analysis (TGA) thermograms and (**b**) differential scanning calorimetry (DSC) curves of the iongels (80 wt % IL) bearing different anions.

The influence of the studied IL anions on the thermal stability of the prepared iongels can be clearly seen in Figure 4a, where the TGA thermograms of the materials with 80 wt % IL content are depicted. The onset and decomposition temperatures of the studied iongels can be ordered in terms of the IL anion’s structure as follows: *T_onset_* [B(CN)_4_]^−^ ≈ [TFSI]^−^ > [C(CN)_3_]^−^ > [FSI]^−^; and *T_dec_* [TFSI]^−^ > [B(CN)_4_]^−^ > [C(CN)_3_]^−^ > [FSI]^−^. 

From Table 1, it can be perceived that both *T_onset_* and *T_dec_* of the iongels are generally lower compared to those of the neat ILs, which can be attributed to the incorporation of PEGDA. For instance, and considering that PEGDA has lower thermal stability when compared to [C_2_mim][TFSI] and [C_2_mim][B(CN)_4_] ILs, the resulting PEGDA–TFSI and PEGDA–B(CN)_4_ iongels exhibit *T_onset_* and *T_dec_* values between those of PEGDA and the respective ILs. However, the opposite trend was found for the PEGDA–C(CN)_3_ iongels that present slightly higher *T_onset_* (330–361 °C) values compared to that of [C_2_mim][C(CN)_3_] IL (324 °C). In this case, it seems that the presence of PEGDA led to the increment of the *T_onset_* values, which is more pronounced when the lowest content (10 wt %) of PEGDA is incorporated in the iongel. Moreover, an odd behavior was observed for the PEGDA–FSI iongels, since the obtained *T_onset_* (229–248 °C) and *T_dec_* (323–344 °C) values are lower when compared not only to those of PEGDA (365 and 396 °C, respectively), but also to the pure [C_2_mim][FSI] IL (294 and 343 °C, respectively). In fact, the different behaviors observed might be a result of specific interactions between the polymer network and the diverse ILs, and thus the thermal stability of the prepared iongels does not always follow a mixing rule.

The thermal properties of the cross-linked PEGDA, neat ILs and respective iongels were also characterized by DSC analysis. The melting (*T_m_*) and glass transition (*T_g_*) temperatures, in the cases where they were observed, are listed in Table 1. The DSC curves of the iongels with 80 wt % IL content are depicted in Figure 4b, while the DSC curves of all the prepared iongels are provided as Supplementary Materials (Figure S6).

Figure 4b shows that the cross-linked PEGDA is a rubbery, amorphous polymer with a single *T_g_*. On the other hand, the neat ILs displayed *T_m_* between −13 and 4 °C, with the only exception being the [C_2_mim][B(CN)_4_] IL since no *T_m_* was observed in the temperature range studied (from −70 to 150 °C). Similarly, no obvious *T_g_* was detected for all the neat ILs (Figure S6). 

Looking at Table 1, it can be seen that some of the prepared iongels showed single *T_g_*, whose values are significantly lower compared to those of only PEGDA. The fact that only one *T_g_* can be observed is associated with the successful blending of the ILs into the cross-linked PEGDA, probably implicating molecular interactions that favor the stability of the resulting systems. The reduction in the *T_g_* values indicates an increase in the flexibility of the polymer chains due to the presence of IL. For instance, the iongels containing different ILs display slightly distinct values of *T_g_* with the same IL content (60 wt %): −43, −47, −50, and −51 °C for the iongels containing [C_2_mim][B(CN)_4_], [C_2_mim][TFSI], [C_2_mim][C(CN)_3_] and [C_2_mim][FSI], respectively, compared with −20 °C of cross-linked PEGDA. The drop in *T_g_* is more pronounced for the PEGDA–60 FSI iongel, meaning that the [C_2_mim][FSI] IL has a stronger plasticizing effect within the PEGDA polymer chains. In addition, the *T_g_* of PEGDA–B(CN)_4_ iongels decreases with increasing IL content (from 60 to 80 wt %), as well as the *T_g_* of the PEGDA–TFSI iongels, which decrease from −47 to −50 °C when the IL content increases from 60 to 70 wt %, indicating the overall increase in polymer chain mobility with increasing IL content. It should be mentioned that, in the case of the iongels, *T_g_* values bellow −51 °C were not detected, probably due to the limitations concerning the operational temperature (−70 °C) of the DSC equipment used in this work. 

Analyzing the DSC curves of the iongels (Figure S6 and Table 1), it can also be seen that melting transitions were not detected in the PEGDA–B(CN)_4_ iongels, which is consistent with the absence of *T_m_* in the corresponding IL. In contrast, melting transitions roughly similar to those of the neat ILs can be observed in the iongels containing [C_2_mim][FSI], [C_2_mim][TFSI] and [C_2_mim][C(CN)_3_]. This observation can be attributed to the presence of free IL domains dispersed in the polymer network, which are not strongly associated with the polymer chains. As evidenced by the increment in the respective enthalpy of melting (*∆H_m_*) values (Table 1), the melting peaks became more intense when the IL content increased from 60 to 90 wt % into the iongels (Figure S6) due to the lower amount of PEGDA. Therefore, the polymer chains were much further apart and the presence of IL-rich phases within the iongel network was more evident in the cases of PEGDA–90 FSI, as well as PEGDA–C(CN)_3_ with 80 and 90 wt % IL content, showing *∆H_m_* of 22, 13 and 33 J g^−1^, respectively. 

### 3.3. Mechanical Properties

**Figure 5:**
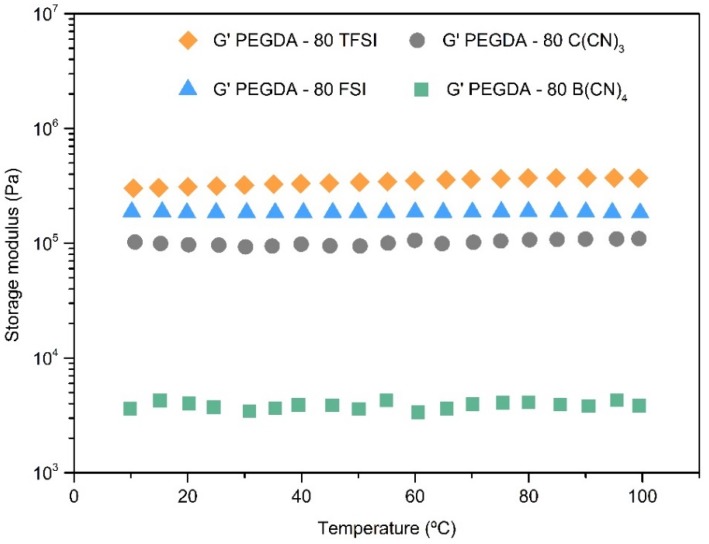
Storage modulus as a function of temperature of the iongels (80 wt % IL) bearing different anions.

The mechanical properties of the iongels were evaluated by DMTA. Specifically, the effect of both the IL content and IL anion structure were assessed, and the cross-linked architecture of the iongels was confirmed. The curves of the storage modulus (Pa) as a function of temperature (°C) of the iongels with 80 wt % IL content are illustrated in Figure 5, while the DMTA analyses of all the prepared iongels are given in Supplementary Materials (Figure S7). It is worth mentioning that it was not possible to perform the DMTA analysis with PEGDA–90 B(CN)_4_ owing to its fragility and, consequently, the easy breakability when handling the sample (Figure 2). Likewise, the sensitivity of the DMTA device prevented a reliable measurements of loss modulus for the iongels with 90 wt % IL content, as well as for the ones containing 80 wt % of the cyano-based ILs. For that reason, only the storage moduli of those iongels are represented in Figure S7.

In general, the iongels unveil a typical rubber-like behavior, as the storage modulus (*G’*) of the materials is above the loss modulus (*G’’*) in the whole range of temperatures studied (Figure S7), confirming a cross-linked network and the solid nature of the free-standing iongels. This behavior is in agreement with what has been reported in the literature for other UV-cross-linked iongels [31,50,51]. In addition, the storage modulus is practically constant between 10 and 100 °C for the majority of the prepared iongels, meaning that their solid-state structures are not destroyed. This is particularly interesting for gas membrane operation, since the iongels maintain their mechanical stability at different temperatures up to 100 °C.

The prepared iongels display values of storage modulus between 3 × 10^3^ and 3 × 10^6^ Pa. As illustrated in Figure S7, the storage modulus significantly decreases when increasing the amount of IL (from 60 to 90 wt %) incorporated into the iongels. This effect is attributed to the fact that network densities decrease as PEGDA content decreases, but the reduction in the storage modulus is not linear. For instance, among the PEGDA–TFSI with 60, 70, 80 and 90 wt % IL content, the storage modulus (at 20 °C) decreased by only 41%, 51% and 58%, respectively while, for the PEGDA–C(CN)_3_ iongels, decays of 81%, 58% and 96% occurred.

As can be seen in Figure 5, the storage modulus decreases in the following order of the IL anion’s structure: [TFSI]^−^ > [FSI]^−^ > [C(CN)_3_]^−^ > [B(CN)_4_]^−^. The same trend was also observed for the iongels comprising other IL contents, the only exception being the ones with 60 wt % IL, since the PEGDA–60 FSI exhibited higher storage modulus (*G’*) values than those of the PEGDA–60 TFSI in the whole temperature range studied (Figure S7a,b). Overall, the iongels made of fluorinated-based anions revealed better mechanical properties when compared to the ones containing cyano-functionalized anions. 

### 3.4. Morphology

**Figure 6:**
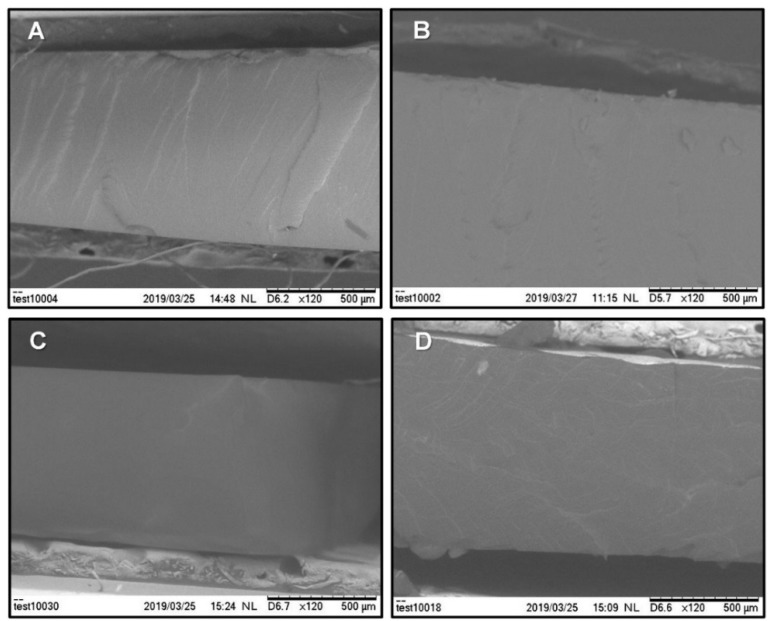
SEM micrographs of fracture cross-section of the iongels containing 80 wt % of the selected ILs: (**A**) [C_2_mim][FSI], (**B**) [C_2_mim][TFSI], (**C**) [C_2_mim][C(CN)_3_] and (**D**) [C_2_mim][B(CN)_4_].

A selection of SEM micrographs of a fracture in the cross-section of the iongels with 80 wt % IL content is shown in Figure 6. The different ILs were successfully incorporated into the PEGDA polymer network. Dense membrane morphologies and defect free structures were obtained, since agglomeration due to phase separation or noticeable deformations were not detected. This is obviously due to the good miscibility between the different ILs and the polymer network. Note that, for the SEM micrographs A and D, it is possible to see some randomly distributed reliefs, which are probably related to plastic deformation during the fracturing process under liquid nitrogen. In general, the prepared iongels are soft and ductile materials, thus it is not easy to fracture their samples and achieve clean cuts.

### 3.5. CO_2_ Solubility

**Figure 7:**
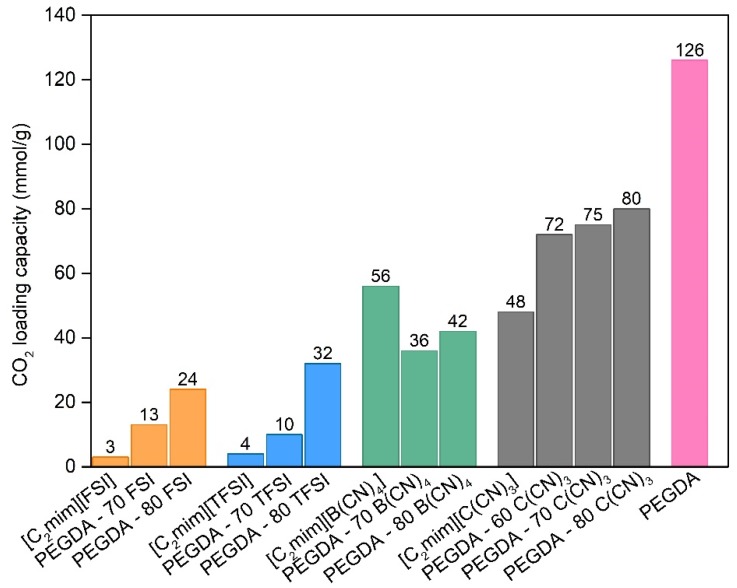
CO_2_ loading capacity (at 30 °C) for the neat ILs, cross-linked PEGDA and different iongels.

Carbon dioxide loading capacity (expressed in mmol of CO_2_ per g of sample) for the different iongels, neat ILs and cross-linked PEGDA were determined by the pressure decay method until its saturation. The values obtained are reported in Figure 7.

For the neat ILs, the CO_2_ solubility follows the trend: [C_2_mim][FSI] < [C_2_mim][TFSI] [C_2_mim][B(CN)_4_] < [C_2_mim][C(CN)_3_]. Even though it is rather difficult to compare the absolute values obtained in this work with those available in the literature due to the different experimental conditions used and the reported units, the trend obtained herein is similar to what has been observed by other authors [52,53,54]. 

The presence of PEGDA in the iongels promoted an increase in CO_2_ uptake when compared with the neat ILs, namely [C_2_mim][FSI], [C_2_mim][TFSI] and [C_2_mim][C(CN)_3_] and this increase is more evident when the IL content increased. However, an odd behaviour was observed for both PEGDA iongels with 70 and 80 wt % [C_2_mim][B(CN)_4_] IL, in which the CO_2_ uptake values obtained are lower compared to that the neat IL. This probably happened due to poor interactions between the polymer network and this IL. In addition, when compared to the work developed by McDanel et al. [22] where cross-linked ionic resins and iongels using imidazolium-based IL monomers were prepared, the solubility values obtained were within the range 0.14 and 1.86 mmol/g, much lower than the ones obtained in the present work. This validates the potential of using the prepared iongels for the separation of CO_2_ from other gases, such as N_2_.

### 3.6. CO_2_/N_2_ Separation Performance

**Figure 8:**
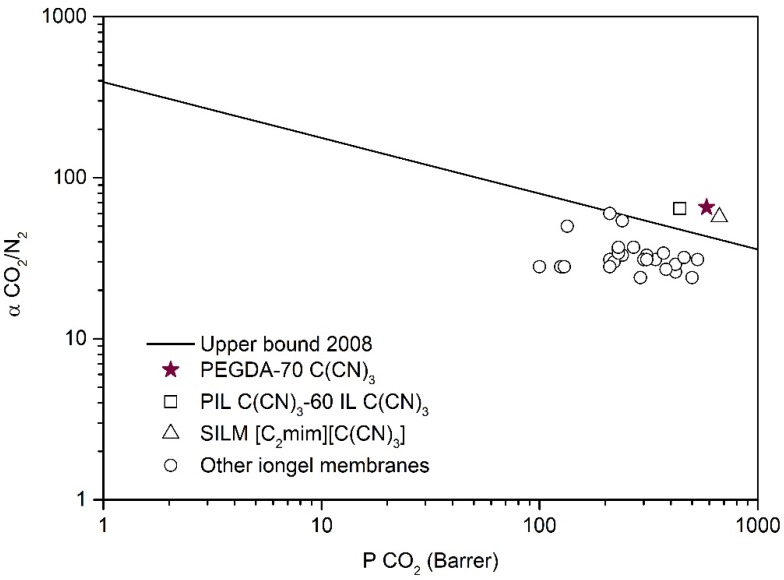
CO_2_/N_2_ iongel ideal selectivity as a functional of CO_2_ permeability. Data are plotted on a log – log scale and the upper bound is adapted from Robeson [55]. The literature values are illustrated for comparison purposes and were taken from: (□) [12], (∆) [37] and (o) [20,22,34,35,36].

Taking into account the CO_2_ solubility values obtained (Figure 7) for the different iongels prepared, as well as the mechanical results obtained by DMTA analysis (Figure S7c), the PEGDA–70 C(CN)_3_ iongel was selected to perform single CO_2_ and N_2_ gas permeability measurements. This iongel was supported on a porous PA membrane filter in order to provide stronger mechanical stability and enable correct gas permeation experiments. Single CO_2_ permeabilities of 583 ± 29 Barrer and ideal CO_2_/N_2_ selectivities of 66 ± 3 were achieved. For comparison purposes, the values obtained in this work and the literature data are depicted in Figure 8, where the CO_2_/N_2_ selectivity is plotted against the permeability of the more permeable gas (CO_2_) in the so-named Robeson plot. 

The CO_2_/N_2_ separation performance of the semi-solid PEGDA–70 C(CN)_3_ iongel membrane is positioned in the same Robeson plot region of the best performing IL-based membranes (Figure 8). For instance, CO_2_ permeabilities of the 667 Barrer and ideal CO_2_/N_2_ selectivities of 57 were obtained for an SILM containing the [C_2_mim][C(CN)_3_] IL [37], while CO_2_ permeabilities of 439 Barrer and ideal CO_2_/N_2_ selectivities of 64 were previously reported for a PIL–IL composite membrane bearing 60 wt % of free [C_2_mim][C(CN)_3_] IL [12]. Therefore, the single-pot methodology employed in this work is not only a promising strategy to overcome the more complicated preparation routes of PIL–IL iongel membranes, but also to achieve improved CO_2_ permeabilities, mainly due to the presence of higher IL contents (> 60 wt %). 

From Figure 8, it can also be observed that the CO_2_/N_2_ separation performance of the PEGDA–70 C(CN)_3_ iongel is superior when compared to those recently reported for other PEG-based cross-linked iongel membranes [34,35,36], probably due to the different cross-linking densities and polymer matrices, as well as the IL used. These results clearly indicate that the combination of a PEGDA monomer (M_n_ 575 g mol^−1^) with high contents of the best performing ILs can lead to enhanced CO_2_/N_2_ separation performances. 

## 4. Conclusions

Iongels based on PEGDA and four ILs, combining the [C_2_mim]^+^ cation and different anions ([TFSI]^−^, [FSI]^−^, [C(CN)_3_]^−^ and [B(CN)_4_]^−^), were developed. The influence of both the IL content and IL anion on the iongels’ thermal and mechanical stability, as well as CO_2_ solubility, was systematically investigated. The iongels were easily prepared in a single-pot by fast (< 1 min) UV-initiated polymerization of PEGDA in the presence of IL. Flexible, transparent, and free-standing iongel membranes were successfully obtained up to 80 wt % IL content. 

The FTIR study confirmed the high extent of the photopolymerization process of acrylate from PEGDA. The DSC analysis revealed lower *T_g_* values for the iongels when compared to that of cross-linked PEGDA, indicating an increase in polymer chain mobility, which is dependent of the IL content, as well as the IL anion’s nature. Although all the prepared iongels have a high thermal stability (*T_onset_* values ranging from 231 to 364 °C) for CO_2_/N_2_ separation processes, the storage modulus (*G’*) significantly decreases from 3 × 10^6^ to 3 × 10^3^ Pa with the amount of IL incorporated. 

The CO_2_-loading capacities of the iongels were found to be higher when compared with the neat ILs, but this increment was more evident when the IL content increased. Furthermore, the PEGDA–70 C(CN)_3_ iongel membrane was able to exceed the CO_2_/N_2_ upper bound limit, surpassing the performance of other PEG-based cross-linked iongel membranes. Actually, in comparison with the previously reported PIL C(CN)_3_ – 60 IL C(CN)_3_, the CO_2_ permeability of the 583 ± 29 Barrer obtained in the PEGDA–70 C(CN)_3_ is superior, whilst keeping similar ideal CO_2_/N_2_ selectivity (66 ± 3), with the additional advantage of its simpler one-pot preparation method. Nevertheless, future improvements to the PEGDA-based iongel mechanical stability should be addressed and will be the subject of our further research. 

## Figures and Tables

**Table 1:** Thermal properties of cross-linked PEGDA, neat ILs and the prepared iongels: onset (*T_onset_*), decomposition (*T_dec_*), melting (*T_m_*) and glass transition (*T_g_*) temperatures, and enthalpy of melting (*∆H_m_*).

Sample	*T_onset_* (°C) ^a^	*T_dec_* (°C) ^b^	*T_m_* (°C)	*T_g_* (°C) ^c^	*∆H_m_* (J g^−1^)
PEGDA	365	396	ND ^f^	−20	-
PEGDA – 60 FSI	248	344	−13	−51	0.7
PEGDA – 70 FSI	229	342	−15	ND	1.1
PEGDA – 80 FSI	231	334	−12	ND	1.4
PEGDA – 90 FSI	240	323	−15	ND	22
[C_2_mim][FSI]	294	343	−11	ND	40
PEGDA – 60 TFSI	361	406	−15	−47	0.4
PEGDA – 70 TFSI	352	418	−14	−50	0.9
PEGDA – 80 TFSI	359	436	−15	ND	1.2
PEGDA – 90 TFSI	342	436	−15	ND	3.0
[C_2_mim][TFSI]	424	463	−13	ND	53
PEGDA – 60 C(CN)_3_	340	378	3	−50	1.0
PEGDA – 70 C(CN)_3_	330	371	4	ND	2.2
PEGDA – 80 C(CN)_3_	334	378	−4	ND	13
PEGDA – 90 C(CN)_3_	361	389	0	ND	33
[C_2_mim][C(CN)_3_]	324	458	4	ND	48
PEGDA – 60 B(CN)_4_	360	392	ND	−43	-
PEGDA – 70 B(CN)_4_	358	393	ND	−51	-
PEGDA – 80 B(CN)_4_	364	401	ND	−50	-
PEGDA – 90 B(CN)_4_	358	410	ND	ND	-
[C_2_mim][B(CN)_3_]	391	427	ND	ND	-

^a^*T_onset_* (onset temperature) defined as the temperature at which the baseline slope changes during the heating. ^b^ *T_dec_* (decomposition temperature) defined as the temperature at 50% weight loss. ^c^ *T_g_* (glass transition temperature) defined as the temperature at the middle point of the glass transition region. ^f^ ND—not detected.

## Supplementary Materials

The following are available online at https://www.mdpi.com/2077-0375/10/3/46/s1, Figures S1–S7: FTIR analysis, TGA thermograms, DSC curves and DMTA analysis of the cross-linked PEGDA iongels containing different amounts of the selected ionic liquids ([C_2_mim][FSI], [C_2_mim][TFSI], [C_2_mim][C(CN)_3_] and [C_2_mim][B(CN)_4_]).

## Abbreviations

*∆H_m_*	Enthalpy of melting
*A*	Membrane area
ATR-FTIR	Total Reflection Fourier transform infrared spectroscopy
Barrer	1 × 10^−10^ cm^3^ (STP) cm cm^−2^ cmHg^−1^ s^−1^
CO_2_	Carbon dioxide
DN	Double network
DSC	Differential scanning calorimetry
DMTA	Dynamic mechanical thermal analysis
*G’*	Storage modulus
*G’’*	Loss modulus
IL	Ionic liquid
*l*	Membrane thickness
N_2_	Nitrogen
*P*	Permeability
PEG	Poly(ethylene glycol)
PEGDA	Poly(ethylene glycol) diacrylate
*P_feed_*	Pressure in the feed compartment
PA	Polyamide
PIL	Poly(ionic liquid)
*p_perm_*	Pressure in the permeate compartment
SEM	Scanning electron microscopy
SILM	Supported ionic liquid membrane
*T*	Time
*T_dec_*	Decomposition temperature
*Tg*	Glass transition temperature
TGA	Thermogravimetric analysis
*T_onset_*	Onset temperature
UV	Ultraviolet
*V_feed_*	Volume of the feed compartment
*V_perm_*	Volume of the permeate compartment
*α*	Ideal selectivity
*β*	Geometric parameter
**Cations**	
[C_2_mim]^+^	1-Ethyl-3-methylimidazolium
[C_4_mim]^+^	1-Butyl-3-methylimidazolium
**Anions**	
[B(CN)_4_]^–^	Tetracyanoborate
[BF_4_]^–^	Tetrafluoroborate
[C(CN)_3_]^–^	Tricyanomethanide
[FSI]^–^	Bis(fluorosulfonyl)imide
[Inda]^–^	Indazole
[PF_6_]^–^	Hexafluorophosphate
[Pro]^–^	Prolinate
[TFSI]^–^	Bis(trifluoromethylsulfonyl)imide
