# Supplementary material for: Influence of Anion Structure on Thermal, Mechanical and CO2 Solubility Properties of UV-Cross-Linked Poly(ethylene glycol) Diacrylate Iongels
Source: Membranes (Basel). 2020 Mar 17;10(3):46. doi: 10.3390/membranes10030046 (PMC7143667; doi:10.3390/membranes10030046)
Supplement: Supplementary file 1 [file membranes-10-00046-s001.pdf]

membranes

MDPI

Article

Supplementary Materials: Influence of Anion Structure on Thermal, Mechanical and CO_2 Solubility Properties of UV-Cross-Linked Poly(ethylene glycol) Diacrylate Iongels

Ana P.S. Martins [1,2], Asier Fdz De Añastro [1], Jorge L. Olmedo-Martínez [1], Ana R. Nabais [3], Luísa A. Neves [3], David Mecerreyes,[1,4] and Liliana C. Tomé [1,*]

[1] POLYMAT, University of the Basque Country UPV/EHU, Joxe Mari Korta Center, Avda. Tolosa 72, 20018 Donostia-San Sebastian, Spain; apd.martins@campus.fct.unl.pt (A.P.S.M.); afernandezdea021@gmail.com (A.F.D.A); jorge.olmedo.martinez@gmail.com (J.L.O.M); david.mecerreyes@ehu.es (D.M)
[2] Instituto de Tecnologia Química e Biológica António Xavier, Universidade Nova de Lisboa, 2780-157 Oeiras, Portugal
[3] LAQV-REQUIMTE, Chemistry Department, Faculdade de Ciência e Tecnologia, Universidade Nova de Lisboa, 2829-516 Caparica, Portugal; a.nabais@campus.fct.unl.pt (A.R.N); lan11892@fct.unl.pt (L.A.N)
[4] Ikerbasque, Basque Foundation for Science, E-48013 Bilbao, Spain
* Correspondence: lilianasofi.carvalho@ehu.eus

Received: 4 February 2020; Accepted: 11 March 2020; Published: 17 March 2020

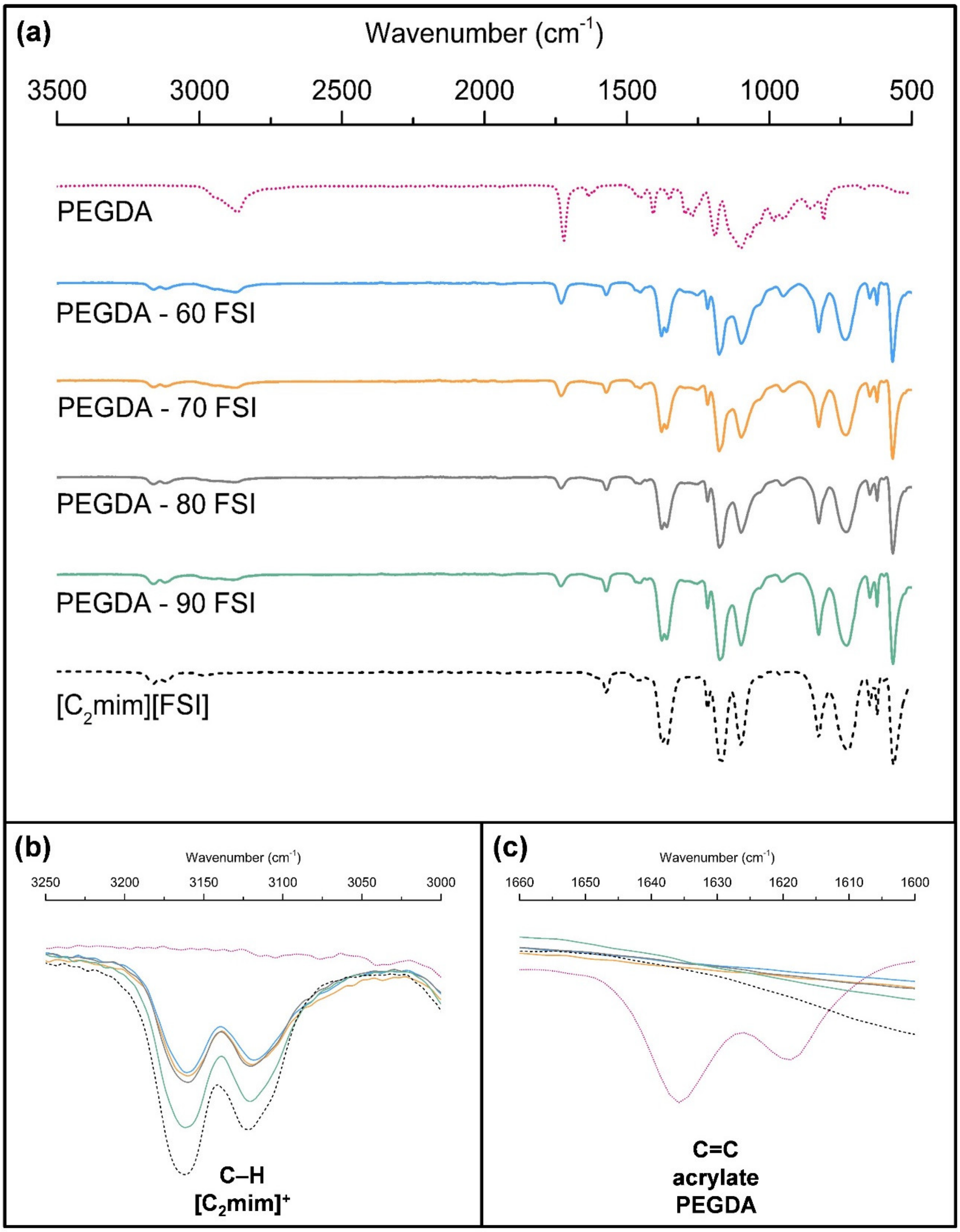

Figure S1. FTIR analysis of the cross-linked PEGDA iongels containing different amounts of [C2mim][FSI]: **(a)** FTIR curves; **(b)** differences in the intensity of the absorption bands associated to the imidazole ring; and **(c)** disappearance of the characteristic absorption bands of acrylate groups.

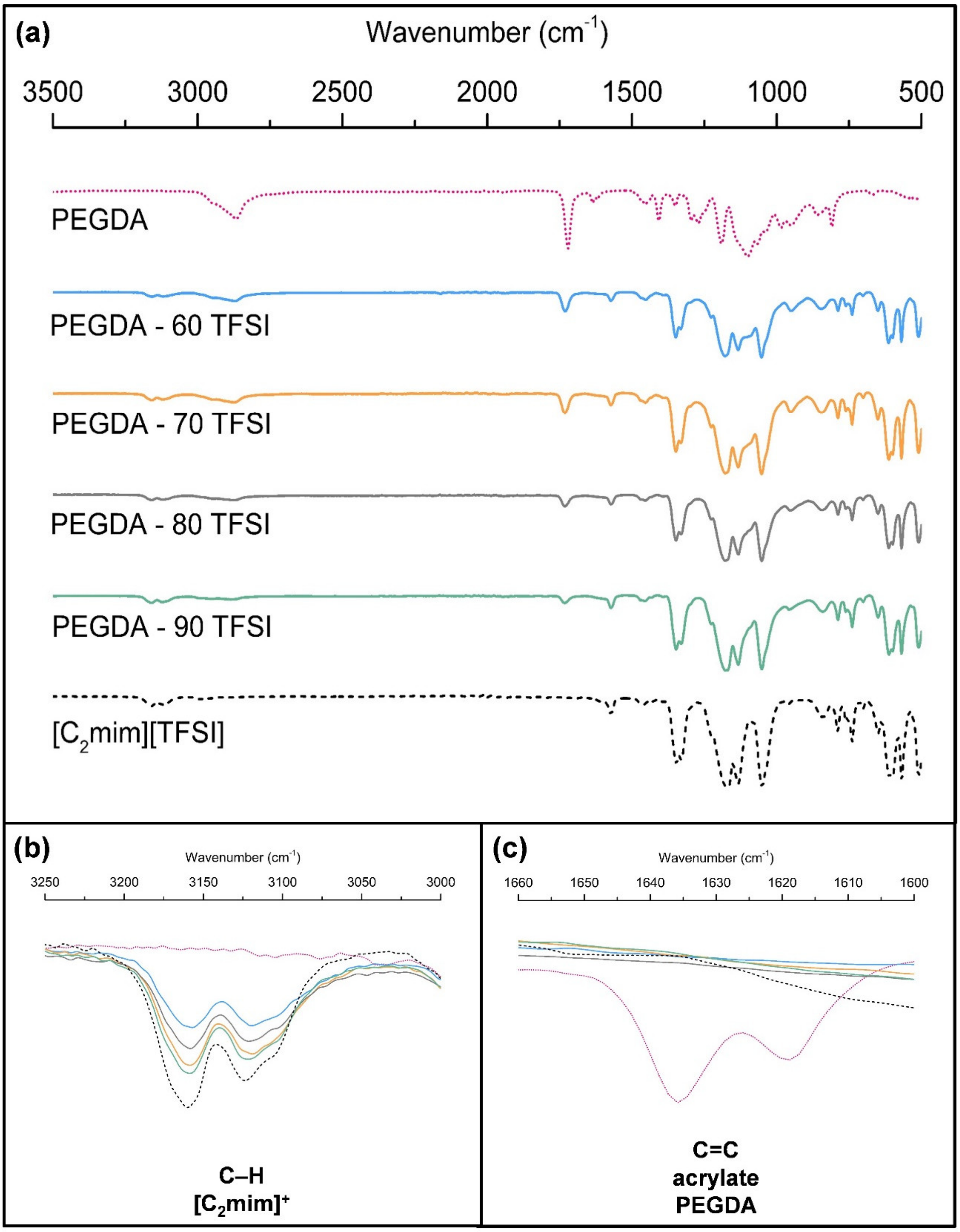

Figure S2. FTIR analysis of the cross-linked PEGDA iongels containing different amounts of [C_2mim][TFSI]: **(a)** FTIR curves; **(b)** differences in the intensity of the absorption bands associated to the imidazole ring; and **(c)** disappearance of the characteristic absorption bands of acrylate groups.

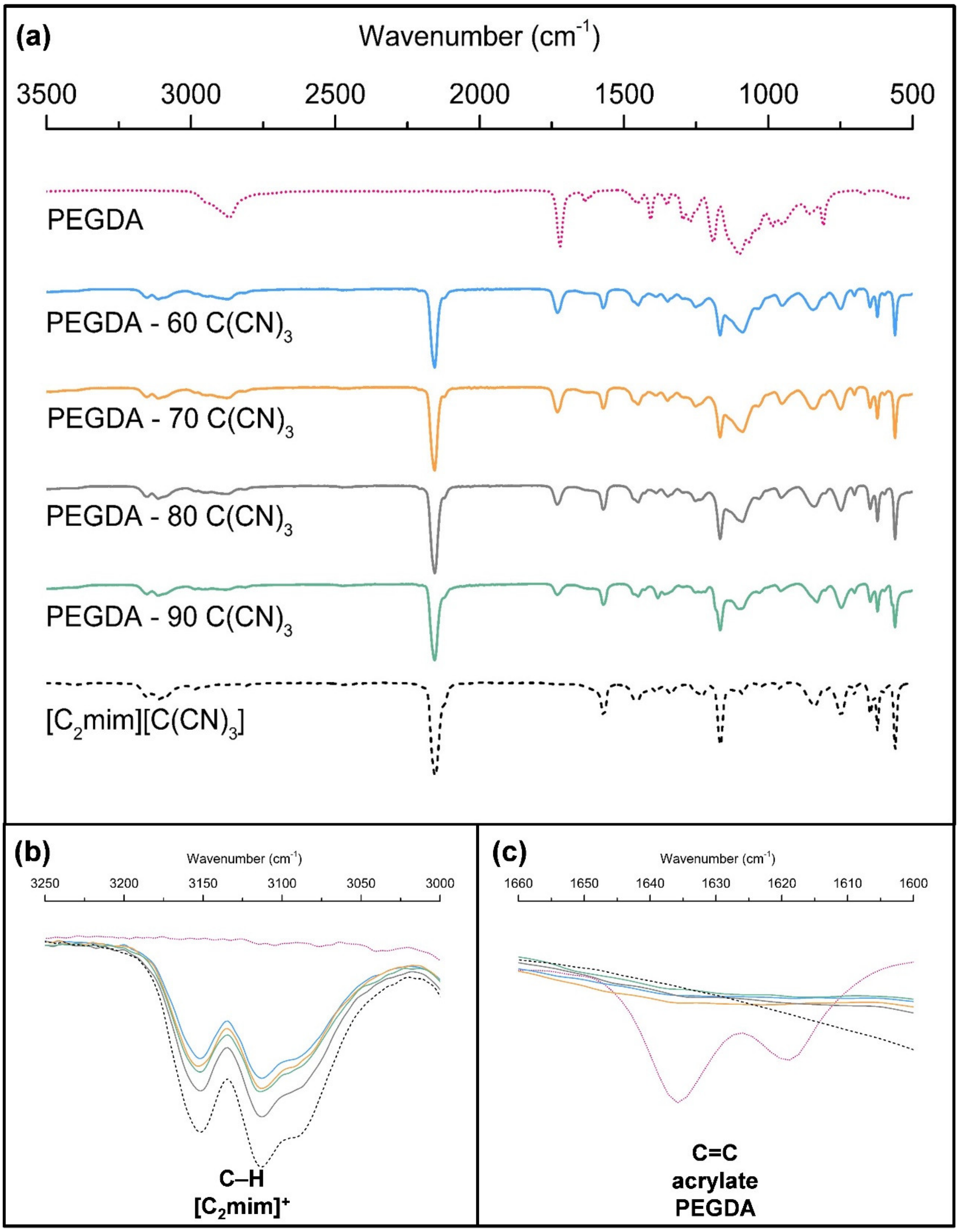

Figure S3. FTIR analysis of the cross-linked PEGDA iongels containing different amounts of [C_2mim][C(CN)$_3$]: **(a)** FTIR curves; **(b)** differences in the intensity of the absorption bands associated to the imidazole ring; and **(c)** disappearance of the characteristic absorption bands of acrylate groups.

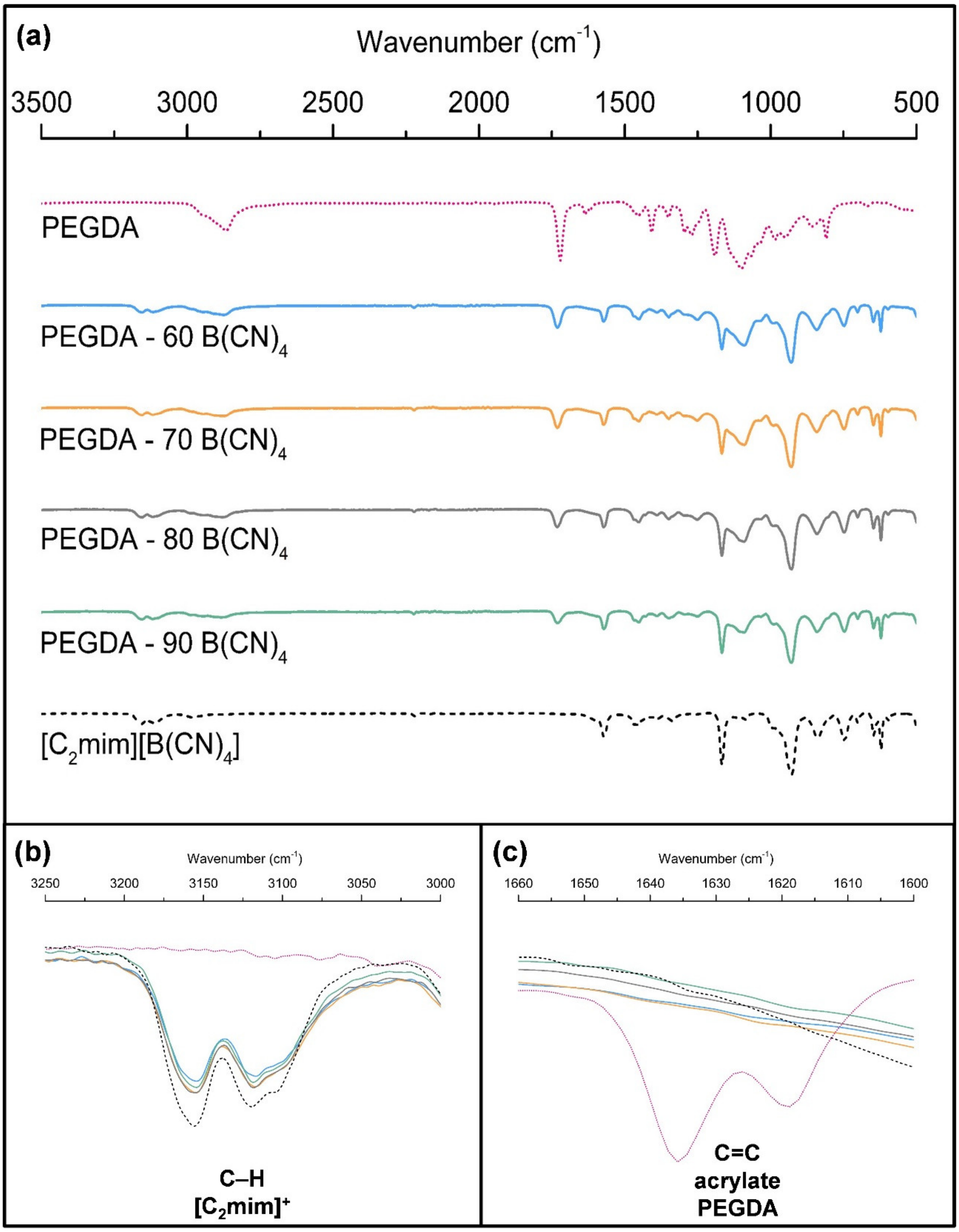

Figure S4. FTIR analysis of the cross-linked PEGDA iongels containing different amounts of $[C_2mim][B(CN)_4]$: **(a)** FTIR curves; **(b)** differences in the intensity of the absorption bands associated to the imidazole ring; and **(c)** disappearance of the characteristic absorption bands of acrylate groups.

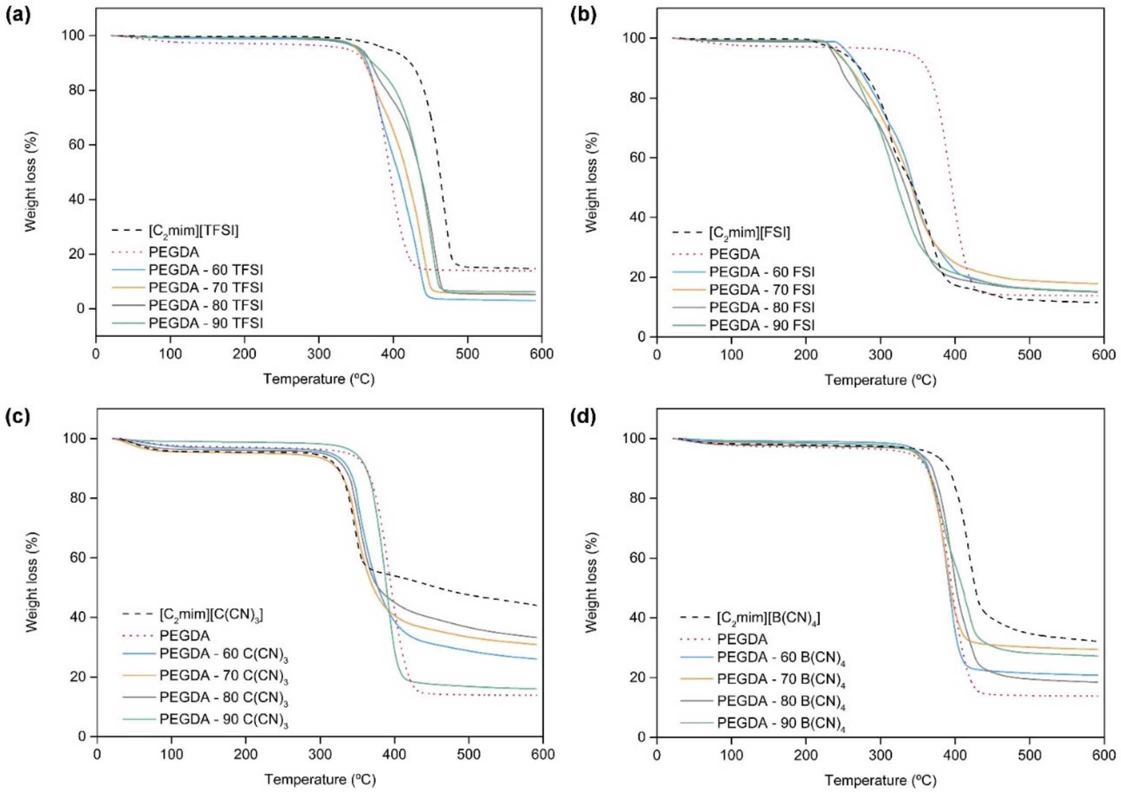

Figure S5. TGA thermograms of the cross-linked PEGDA iongels containing different amounts of the selected ionic liquids: **(a)** [C_2mim][TFSI], **(b)** [C_2mim][FSI], **(c)** [C_2mim][$C(CN)_3$] and **(d)** [C_2mim][$B(CN)_4$].

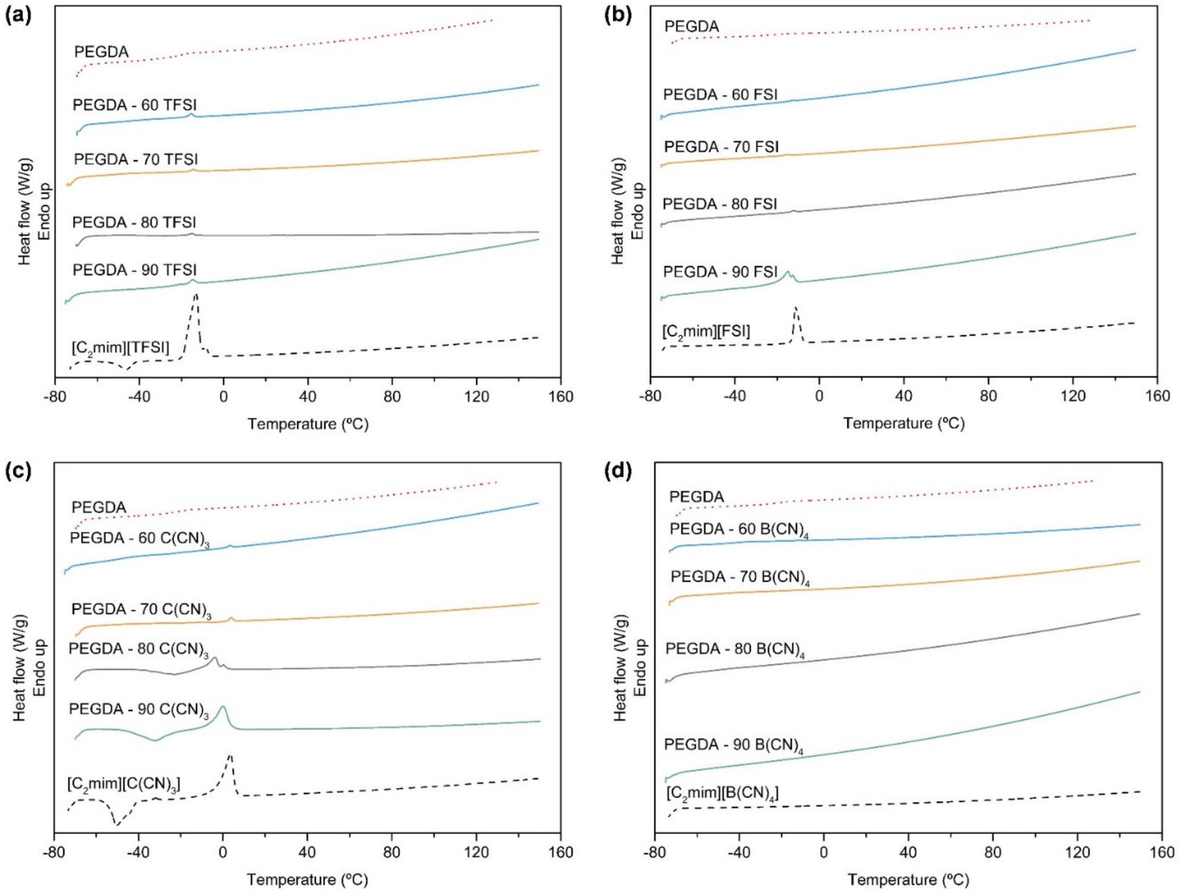

Figure S6. DSC curves of the cross-linked PEGDA iongels containing different amounts of the selected ionic liquids: **(a)** [C_2mim][TFSI], **(b)** [C_2mim][FSI], **(c)** [C_2mim][$C(CN)_3$] and **(d)** [C_2mim][$B(CN)_4$].

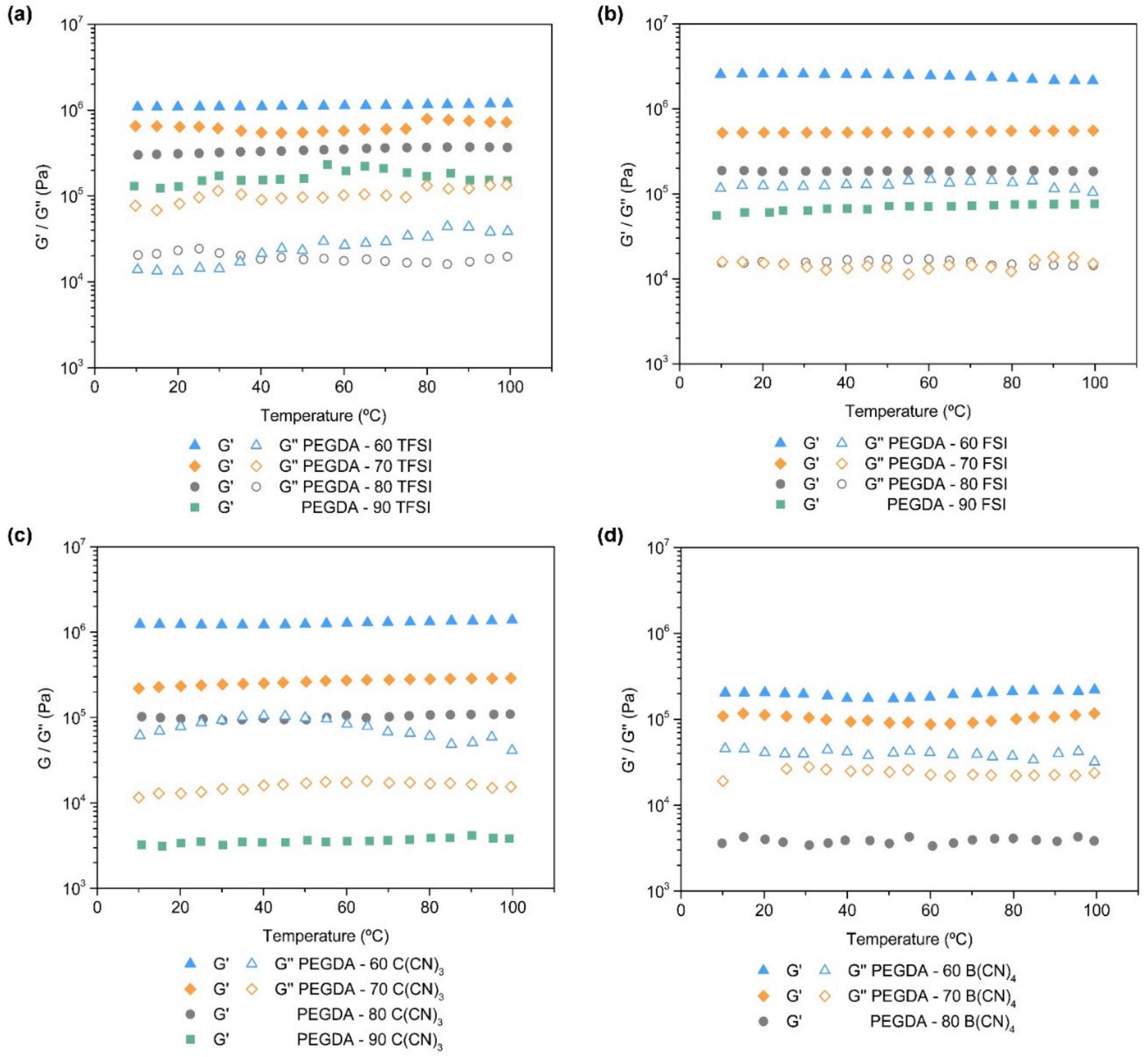

Figure S7. DMTA analysis of the cross-linked PEGDA iongels containing different amounts of the selected ionic liquids: **(a)** [C_2mim][TFSI], **(b)** [C_2mim][FSI], **(c)** [C_2mim][C(CN)$_3$] and **(d)** [C_2mim][B(CN)$_4$].

© 2020 by the authors. Submitted for possible open access publication under the terms and conditions of the Creative Commons Attribution (CC BY) license (http://creativecommons.org/licenses/by/4.0/).
